# Annual Meeting of the Erie County Medical Society

**Published:** 1865-01

**Authors:** 


### EDITORIAL DEPARTMENT.

### ANNUAL MEETING OF THE ERIE COUNTY MEDICAL SOCIETY.

The Annual Meeting of the Erie County Medical Society was held January 10th, and was quite largely attended; the severe storm not preventing members from the towns from being present in considerable numbers. The Annual Address was given by Dr. Wetmore of Buffalo. It was complimented by vote of thanks, and that a copy be furnished for publication in the *Buffalo Medical and Surgical Journal.*

Dr. C. C. Wyckoff was elected delegate to the State Medical Society for an unexpired term, made vacant by resignation. By this arrangement we have no doubt this Society will be represented at the next meeting of the State Medical Society.

In the afternoon a few members convened promptly at the time of adjournment and went through the form of electing officers for the ensuing year. The usual order of promotion was ignored in some respects, but the rolls were filled by worthy names, which is sufficient report, to make of a matter in which a great majority of the Society manifest no interest whatever.

Prof. Joseph Wiel, of Germany, who is on a visit to this country, was elected a corresponding member of the Society, and invited to participate in the meeting. An invitation was also given him to exhibit before the Society the laryngoscope and show its uses; and Wednesday evening was designated for this purpose.

At the time appointed a large number of the profession of the city were present, who manifested great interest and gratification in the opportunity thus afforded of seeing the larynx, vocal cords, etc., of a patient who had manifest laryngeal disease, which was pointed out distinctly. Prof. Wiel showed great familiarity with the working of the instrument, and skill in detecting any morbid changes which had taken place within the range of investigation. It was not however attempted to demonstrate the condition of disease so much as to show the scope and value of this mode of examination—to convince of the value of the laryngoscope in diagnosing and treating diseases of the fauces, larynx and trachea.

After the illustrations, the informal meeting was organized by calling Dr. Josiah Barnes to the Chair, and inviting Dr. John Hauenstein to act as Secretary.

Dr. Miner remarked that his object in asking for an organization of the meeting, was for the purpose of affording the Society opportunity of expressing the pleasure and gratification afforded by the illustrations presented by Dr. Wiel, and of extending to him in proper terms its thanks for the successful efforts he had made to demonstrate the practical workings and uses of an instrument, which, in his skilled hands, at least, was seen capable of showing conditions of disease in situations wholly undiscoverable until brought to light by the invention of this instrument, and the enlightened efforts of men like himself, who had devoted their time, and thought, to its practical workings and benefits.

For the purpose of bringing these sentiments in form for the action of the Society, would *move:* That the thanks of the Medical Society of Erie County be presented Prof. Wiel for the successful and highly gratifying illustrations he had given with the laryngoscope, with hearty good wishes for his safe return to his native country, and continued success in discovering and removing conditions of disease, which have hitherto, in great degree, remained unseen and unhealed.

This motion was unanimously adopted.

Prof. Wiel *replied*, expressing thanks for the honors the Society had conferred upon him, and his pleasure in meeting so many of the members who had interest in his illustrations of the uses of the laryngoscope.

